# Aiming for Study Comparability in Parkinson's Disease: Proposal for a Modular Set of Biomarker Assessments to be Used in Longitudinal Studies

**DOI:** 10.3389/fnagi.2016.00121

**Published:** 2016-05-27

**Authors:** Stefanie Lerche, Sebastian Heinzel, Guido W. Alves, Paolo Barone, Stefanie Behnke, Yoav Ben-Shlomo, Henk Berendse, Bastiaan R. Bloem, David Burn, Richard Dodel, Donald G. Grosset, Geraldine Hipp, Michele T. Hu, Meike Kasten, Rejko Krüger, Inga Liepelt-Scarfone, Walter Maetzler, Marcello Moccia, Brit Mollenhauer, Wolfgang Oertel, Benjamin Roeben, Uwe Walter, Karin Wirdefeldt, Daniela Berg

**Affiliations:** ^1^Center of Neurology, Department of Neurodegeneration and Hertie-Institute for Clinical Brain Research, University of TuebingenTuebingen, Germany; ^2^German Center for Neurodegenerative Diseases, University of TuebingenTuebingen, Germany; ^3^Norwegian Centre for Movement Disorders, Stavanger University HospitalStavanger, Norway; ^4^Neuroscience Section, Center for Neurodegenerative Diseases (CEMAND), Department of Medicine and Surgery, University of SalernoSalerno, Italy; ^5^Department of Neurology, Saarland University HospitalHomburg, Germany; ^6^School of Social and Community Medicine, University of BristolBristol, UK; ^7^Department of Neurology and Neuroscience Campus Amsterdam, VU University HospitalAmsterdam, Netherlands; ^8^Department of Neurology, Radboud University Medical Center, Donders Institute for Brain, Cognition, and BehaviorNijmegen, Netherlands; ^9^Institute of Neuroscience, Newcastle UniversityNewcastle, UK; ^10^Department of Neurology, Philipps-Universität MarburgMarburg, Germany; ^11^Institute of Neurological Sciences, Queen Elizabeth University HospitalGlasgow, UK; ^12^Clinical and Experimental Neuroscience, Luxembourg Centre for Systems Biomedicine, University of LuxembourgBelval, Luxembourg; ^13^Centre Hospitalier LuxembourgLuxembourg, Luxembourg; ^14^Nuffield Department of Clinical Neurosciences, Oxford Parkinson's Disease Centre, University of OxfordOxford, UK; ^15^Institute of Neurogenetics, University of LübeckLübeck, Germany; ^16^Paracelsus-Elena-KlinikKassel, Germany; ^17^University Medical Center, Georg-August-Universität GöttingenGöttingen, Germany; ^18^Department of Neurology, University of RostockRostock, Germany; ^19^Department of Epidemiology and Biostatistics, Karolinska InstitutetStockholm, Sweden; ^20^Department of Clinical Neuroscience, Karolinska InstitutetStockholm, Sweden; ^21^Department of Neurology, Christian-Albrechts UniversityKiel, Germany

**Keywords:** Parkinson's disease, marker, harmonization, cohort studies

## Introduction

Parkinson's disease (PD) is an example for a complex field of research, which is driven by the multifactorial etiology, the heterogeneity in phenotype and the variability in disease progression, as well as the presence of a long pre-diagnostic period, called prodromal PD, lasting up to decades (Postuma et al., 2010). The very slow, so far inevitably progressive, neurodegenerative process and the multidimensional heterogeneity of symptoms in kind (motor and non-motor), time of onset and speed of progression call for prediction markers and progression markers to understand the onset of neurodegeneration and its course. These markers would also help to establish endpoints for neuroprotective treatment strategies aiming to modify disease progression. Because of the complexity, heterogeneity, and the progressive nature of PD, such predictive and progression markers can only be identified in large cohorts and in studies with a longitudinal design. A considerable number of longitudinal cohort studies in PD patients, as well as in individuals at risk, are currently being performed, and extensive effort has gone into the characterization of the individuals assessed. Although each study has its own value and merits, many important research questions cannot be answered as the numbers of participants are too small (e.g., when studying conversion to PD in at-risk populations). Moreover, the pivotal combination of data and findings across studies is hampered by the lack of comparability of symptoms/factors that are being assessed and the specific assessments that are being applied. Therefore, a common approach is needed to enable harmonization and combination of data across studies to define and validate predictive and progression markers.

Based on the need for harmonized assessments of symptoms/markers in PD, the working group: *Harmonization of biomarker assessment in longitudinal cohort studies in Parkinson's Disease* (BioLoC-PD) of the Joint Programme for Neurodegenerative Diseases (JPND), set out to develop an assessment battery that includes the most useful clinical, laboratory, and brain imaging assessments for (longitudinal) studies in PD.

We here describe the result of the process to find a way to harmonize assessments across studies and propose a modular set of biomarker assessments agreed upon by the group of experts who were included in the working group (all authors of this manuscript).

## Materials and methods

As a first step, information about the design, markers, and assessments of 21 ongoing cohort studies in various phases of PD represented by members of the JPND working group were collected using a detailed questionnaire. These data served as a basis for the project. Detailed results have been reported previously in Lerche et al. (2015). In a second step, a systematic literature search on assessments and markers in the prodromal and clinical phases of PD was conducted to determine the most useful predictive and progression markers in PD. The outcome of this literature search was combined with the study information derived from the BioLoC-PD studies and the expert clinical knowledge of the principal investigators in their clinical routine. This information were used to establish the proposed assessment battery. A common modular set of biomarker assessments was defined that includes a basic module and different modules for extension, in order to account for the differences in research focus between studies.

## Results

### Composition of a modular set of biomarker assessments

**Figure 1:**
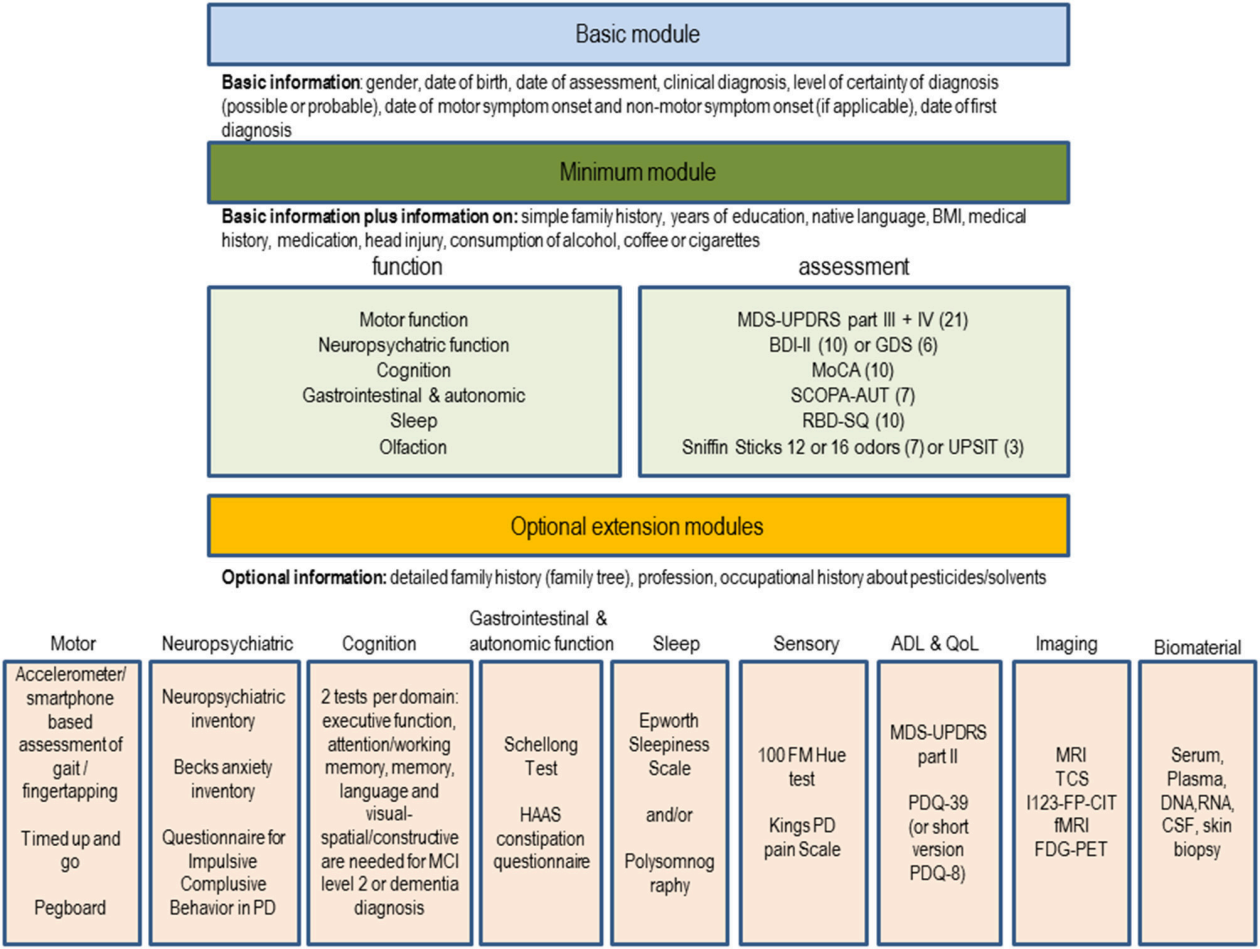
**Suggestion for a modular set of biomarker assessments to be used in longitudinal studies in PD**. The number of studies using the assessment in the BioLoC-PD consortium are given in brackets.

Based on the analyses, the JPND BioLoC-PD working group suggests the following three-level modular assessment battery to be implemented in new and whenever possible ongoing longitudinal studies for PD (Figure 1). The set comprises a **basic** module (demographics, diagnosis, etc.), a **minimum** function and assessment module and several **optional extension** modules.

The **basic** module is meant to be applied to all participants in longitudinal studies in PD. It may also be applied to existing registers and patients seen routinely in outpatient clinics irrespective of whether they are currently recruited for a longitudinal study. It may also be used for retrospective analyses or identification of potential eligible participants for future randomized controlled trials.

The **minimum** module is suitable for all individuals participating in at risk, prodromal, and clinical longitudinal studies of PD. The functions of the minimum module are in a descending order (sorted by their use within the BioLoC-PD working group). Functions used in all (risk, prodromal, and clinical) PD studies are at the beginning of each of the lists (in the minimum and extension modules). Modes of assessment of these symptoms are based on the frequency and applicability within studies (easy to apply assessments, which still provide sufficient information were preferred). Each of the assessments suggested for the minimum module (Figure 1) takes no more than 10 min, depending on the cognitive capacity of the study participant. For some functions (neuropsychiatric and olfactory), two optional assessments are suggested from which one can be chosen. This is because they are used in almost equal proportion in ongoing studies and are similarly tolerable for the participants. For the neuropsychiatric function we recommend to use the BDI in prodromal studies and in early stage PD studies and the GDS in late stage PD studies. For olfaction two assessments are given because they are used in almost equal proportion in ongoing studies are similarly tolerable for the participants. However, the first mentioned suggestion, is slightly preferred by the BioLoC-PD consortium (used more often). Regarding the use of the MMSE or MoCA, we found that the MMSE is more often applied in existing studies but for new studies we clearly recommend to use the MoCA.

The **optional extension** module can be applied to evaluate study participants in more detail. The selection of the additional function modules depends on the main research focus of the study, on the number of study participants and on the available staff and finance. Optional modules can be applied to interesting sub-groups of participants, if financial or pragmatic/practical factors hinder administration to the whole cohort. The assessments included in the extension modules were chosen based on their implementation in the ongoing BioLoC-PD studies. For each subdomain within the extension modules, the most commonly used assessment is suggested in addition to the minimum module (e.g., motor → gait and balance → accelerometer). In case of the imaging module the methods are in a descending order with the one on the top preferred by the BioLoC-PD consortium.

### Application of the modular set of biomarker assessments

We propose, that each study should as a minimum requirement collect the data specified in the basic module. The basic module is valuable also for genetic or other non-clinical analyses. Once individuals are clinically examined, several motor and non-motor domains should be covered, as suggested in the minimum module which also comprises additional data about medical history. Finally, according to the main research aim of the study, different extension modules can be added.

In general, we provide researchers with suggestions for specific assessment tools/scales to allow comparison across studies. For the cognition module, however, it is less important which assessments are used rather, it is important to take a minimum of two tests per domain for a sensitive and specific diagnosis of dementia and Mild Cognitive Impairment (MCI) level II (Goldman et al., 2015). For cognitive analyses, a comparison of studies with different assessments will then be possible by comparing the domain z-scores (Aarsland et al., 2010), equipercentile or item response theory modeling or by using existing conversion algorithms (e.g., conversion between the MMSE and MOCA tests (Lawton et al., 2016)). A list of neuropsychological tests suitable for the optional cognitive extension module can be found in Goldman et al. (2015) and Litvan et al. (2012).

## Discussion

The inventory of assessments used in ongoing longitudinal studies within the working group revealed that there is a consensus about the functions/domains that should be assessed in PD cohort studies, but not about the nature of specific assessments used. The variability in the choice of the assessments may be explained by a number of different factors: (i) Not all scales/questionnaires are available and validated in all languages. (ii) Study designs vary with regard to outcome variables which influences the choice of assessments. (iii) Some assessments require more resources than others (more time-consuming, more costly or requiring trained staff members), which also influenced the selection and composition of the selected assessment battery. (iv) Advances in knowledge about assessments and biomarkers have led to revision or expansion of assessments after the respective study was initiated. (v) Preference for a specific assessment based on previous experience of the individual researchers involved.

With our proposed modular set of biomarker assessments, we propose a concept by which we hope to overcome the problem of data comparison due to lack of harmonization and set the stage for broad data sharing, joint data analyses and acceleration of biomarker research.

## Author contributions

SL, SH, GA, PB, SB, YBS, HB, BRB, DBu, RD, DG, GH, MH, MK, RK, ILS, WM, MM, BM, WO, BR, UW, KW, DBe substantial contributed to the conception and design of the work; SL, SH, DBe drafted the work; GA, PB, SB, YBS, HB, BRB, DBu, RD, DG, GH, MH, MK, RK, ILS, WM, MM, BM, WO, BR, UW, KW revised the work critically for important intellectual content; SL, SH, GA, PB, SB, YBS, HB, BRB, DBu, RD, DG, GH, MH, MK, RK, ILS, WM, MM, BM, WO, BR, UW, KW, DBe gave their final approval to the version to be published; SL, SH, GA, PB, SB, YBS, HB, BRB, DBu, RD, DG, GH, MH, MK, RK, ILS, WM, MM, BM, WO, BR, UW, KW, DBe agreed to be accountable for all aspects of the work.

## Funding

The work was funded by the EU Joint Programme—Neurodegenerative Disease Research (JPND) program (BMBF No:01ED1410).

### Conflict of interest statement

The authors declare that the research was conducted in the absence of any commercial or financial relationships that could be construed as a potential conflict of interest.

## Abbreviations

ADL: Activities of daily living
BDI-II: Beck depression inventory version II
CSF: Cerebrospinal fluid
I^123^-FP-CIT: ^123^I-N-ω-fluoropropyl-2β-carbomethoxy-3β-(4-iodophenyl)nortropane)
DNA: Deoxyribonucleic acid
FDG-PET: fluorodeoxyglucose positron emission tomography
fMRI: functional magnetic resonance imaging
GDS: Geriatric depression scale
HAAS: Honolulu Asia Aging study
MDS-UPDRS: Movement Disorder Society—Unified Parkinson's Disease Rating Scale
MMSE: Mini-Mental State Examination
MoCA: Montreal Cognitive Assessment
MRI: magnetic resonance imaging
PD: Parkinson's Disease
PDQ-39: Parkinson's Disease Questionnaire-39 items
QoL: Quality of Life
RBD-SQ: Rapid eye movement sleep behavior disorder screening questionnaire
RNA: Ribonucleic acid
SCOPA: Scales for Outcome in Parkinson's Disease
TCS: transcranial sonography.
